# MALDI imaging mass spectrometry: statistical data analysis and current computational challenges

**DOI:** 10.1186/1471-2105-13-S16-S11

**Published:** 2012-11-05

**Authors:** Theodore Alexandrov

**Affiliations:** 1 Center for Industrial Mathematics, University of Bremen, Bibliothekstr. 1, 28359 Bremen, Germany; 2 Steinbeis Innovation Center for Scientific Computing in Life Sciences, Richard-Dehmel-Str. 69, 28211 Bremen, Germany; 3 Skaggs School of Pharmacy and Pharmaceutical Sciences, University of California San Diego, 9500 Gilman Drive, La Jolla, CA 92093, USA

## Abstract

Matrix-assisted laser desorption/ionization time-of-flight (MALDI-TOF) imaging mass spectrometry, also called MALDI-imaging, is a label-free bioanalytical technique used for spatially-resolved chemical analysis of a sample. Usually, MALDI-imaging is exploited for analysis of a specially prepared tissue section thaw mounted onto glass slide. A tremendous development of the MALDI-imaging technique has been observed during the last decade. Currently, it is one of the most promising innovative measurement techniques in biochemistry and a powerful and versatile tool for spatially-resolved chemical analysis of diverse sample types ranging from biological and plant tissues to bio and polymer thin films. In this paper, we outline computational methods for analyzing MALDI-imaging data with the emphasis on multivariate statistical methods, discuss their pros and cons, and give recommendations on their application. The methods of unsupervised data mining as well as supervised classification methods for biomarker discovery are elucidated. We also present a high-throughput computational pipeline for interpretation of MALDI-imaging data using spatial segmentation. Finally, we discuss current challenges associated with the statistical analysis of MALDI-imaging data.

## Introduction

In the last decade, matrix-assisted laser desorption/ionization-time of flight (MALDI-TOF) imaging mass spectrometry (IMS), also called MALDI-imaging [1], has seen incredible technological advances in its applications to biological systems [2-7]. While innovative ten years ago, applications to human or animal tissues are now fairly routine with established protocols already in place. New types of samples are continuously being analyzed (e.g. bacterial thin films [3], whole animal body sections [8], plant tissues [5], polymer films [9], and many more) with the main focus on proteomics. Although new IMS techniques are being introduced every year, our recent review [2] shows that MALDI-imaging plays the leading role in the new, rapidly developing field of IMS-based proteomics.

This paper consists of two parts. Firstly, we outline computational methods for MALDI-imaging data analysis with the emphasis on multivariate statistical methods, discuss their pros and cons, and give recommendations on their application. We hope to guide molecular biologists and biochemists through the maze of existing computational and statistical methods. While this paper does not elucidate the basics of existing methodologies, we try to give clear and concise recommendations on when certain methods should be applied. Secondly, we discuss current computational and statistical challenges in analyzing MALDI-imaging data. MALDI-imaging is a relatively new field with only a limited amount of laboratories performing data acquisition, although this number grows rapidly. Presently, this field has a high entry barrier for a computational scientist, since only a few datasets are publicly available. In addition, computational results are normally presented in proteomics or mass spectrometry journals, there fore the computational and statistical challenges are not known in the statistical or bioinformatic communities. We hope that the second part of this paper will attract scientists from these communities to contribute to the fascinating field of computational IMS.

As the field of MALDI-imaging is constantly evolving, novel MALDI-based techniques were recently introduced such as 3D MALDI-imaging [10], MALDI-FTICR- [11] or MALDI-Orbitrap-imaging [12]; however, this paper focuses primarily on conventional MALDI-imaging using a TOF mass analyzer. We do not consider computational methods developed for secondary ion mass spectrometry (SIMS) [13], another leading IMS technique, mainly because SIMS is not used in proteomic analysis with its mass range limited to below 1.0-1.5 kDa. Other emerging IMS techniques such as desorption electrospray ionization (DESI) [14], laser ablation inductively coupled plasma mass spectrometry (LA-ICP-MS) [15], or nanostructure-initiator mass spectrometry (NIMS) [16], are not considered either. In general, all computational methods discussed in this paper can be applied or are already applied (such as PCA in the context of SIMS, see later in the text) to all mentioned IMS techniques. Although we tried to consider only computational methods available in existing software packages, some methods require in-house implementation.

## MALDI imaging mass spectrometry

**Figure 1:**
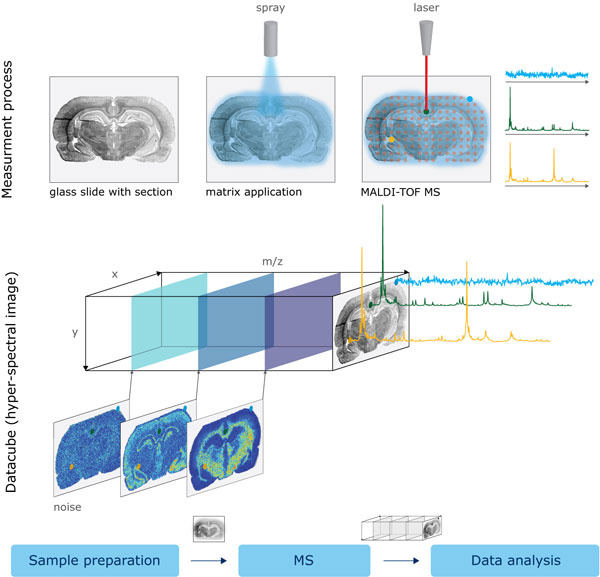
**MALDI-imaging data acquisition workflow**. MALDI-imaging data acquisition workflow and data representation as a datacube or a hyperspectral image with spatial coordinates *x *and *y *and with the mass spectral coordinate *m/z*. For every pair of coordinates (*x, y*) one gets a mass spectrum, for every *m/z*-value one gets an *m/z*-image. The so-called jet colormap from blue (lowest intensity) to yellow to red (highest intensity) was used for *m/z*-images.

Matrix-assisted laser desorption/ionization-time of-flight imaging mass spectrometry, also called MALDI-imaging, emerged in the late 1990s [1,17] and has opened new horizons for application of mass spectrometry in biology and medicine [18]. Once a sample is prepared for analysis (that involves mounting of tissue section, plant leaf or thin agar layer onto a MALDI target plate followed by matrix application), MALDI-imaging mass spectrometry measures mass spectra at discrete spatial points, providing a so-called datacube or hyperspectral image, with a mass spectrum measured at each pixel; see Figure 1. A mass spectrum represents the relative abundances of ionizable molecules with various mass-to-charge (*m/z*) values, ranging for MALDI-TOF-IMS from several hundred *m/z *up to a few tens of thousands *m/z*. An *m/z*-value in MALDI mass spectrometry is usually interpreted as the molecular mass, since ions with a charge of +1 prevail. An intensity of a spectrum at an *m/z*-value represents the relative abundance of a compound with this *m/z*-value. Although MALDI is not a quantitative technique, it can to some extent be used for semi-quantitative comparisons based on the relative abundance of molecules within a spectrum or, after normalization of spectra (more on it later), between spectra [19].

A state of the art MALDI-imaging dataset comprises a huge amount of spectra (usually 5,000-50,000 spectra) with each raw spectrum representing intensities measured at a large number (usually 10,000-100,000) of small *m/z*-bins and describing up to hundreds of different molecules. For any given *m/z*-value, the signal intensity at this *m/z*-value across all collected spectra can be visualized as a pseudo-colored image where each pixel is colored according to its spectrum intensity (sometimes called as a heat map), which we call an *m/z*-image. Definitely, understanding and interpreting such a multitude of spectra or *m/z*-images requires computational data mining methods. Although a dataset can be mined manually, this is a tedious work. Moreover, manual mining normally results in a few - sometimes arbitrarily selected - ions of interest, neglecting the major part of information represented in the IMS dataset.

An ultimate aim of processing, both manual and automated, of a MALDI-imaging dataset is to find *m/z*-values which correspond to ions of interest. These ions may be specific to a spatial region, e.g. be well co-localized with an anatomical region, or express difference between two spatial regions of one sample or between two different samples, e.g. be discriminative for a tumor region as compared with a control region. MALDI-imaging, as a non-targeted and label-free proteomic technique, delivers information about the wide range of molecules present in a sample and is well suited for discovery studies, e.g. for biomarker discovery. Computational methods are of special importance in discovery studies because manual data examination normally results in only a few - sometimes arbitrarily selected - ions. Such incomplete identification can undermine discovery. Once ions of interest are revealed with MALDI-imaging, they can be identified using MS-based proteomics identification methods; for a short review of identification strategies used in combination with MALDI-imaging, see [20].

For a broad review of technological principles and protocols used in IMS and, particularly, in MALDI-imaging, see the recent issue of Methods in Molecular Biology devoted to IMS [21]. Moreover, see recent surveys [2,22,23] for a mass spectrometric perspective and [3] for a microbiology perspective.

## Computational methods

We have structured this section by grouping computational methods according to the tasks they perform: firstly, pre-processing of spectra, then unsupervised data mining methods which can be used for preliminary data examination, then supervised classification applied e.g. in biomarker discovery. A typical MALDI-imaging study results in a set of ions of interest, which are visualized as *m/z*-images corresponding to their *m/z*-values. In the last subsection, we discuss visualization of such images.

### Pre-processing

A MALDI-imaging dataset represents a set of mass spectra with two spatial coordinates *x *and *y *assigned to each spectrum. In the current practice, the pre-processing of MALDI-imaging mass spectra does not differ much from spectra pre-processing in the conventional MALDI-MS of dried droplets and includes (1) normalization, (2) baseline correction, and, optionally, (3) spectra smoothing and (4) spectra recalibration. Standard and well-known MALDI-MS pre-processing methods can be applied to imaging data. For a discussion of mass spectra pre-processing from the MALDI-imaging perspective, see [24].

An important part of MALDI-imaging data pre-processing is the spectra normalization, i.e. scaling each spectrum up to some factor for a better intercomparison of intensities between different spectra. A standard method is the so-called total ion count (TIC) normalization, where for a spectrum its TIC (the sum of all intensities) is calculated and then all spectrum intensities are divided by the TIC value. Although there are still debates on this topic, recent extensive study [25], where TIC and five other normalization methods were considered, demonstrated the need for normalization. TIC is the most popular method and is recommended in general. For more careful analysis, Deininger et al. [25] recommends to consider either TIC or median normalization and to select the proper method by means of visual examination of exemplary *m/z*-images after normalization.

Another pre-processing method, which is sometimes considered separately from the traditional preprocessing methods listed above, is the peak picking, i.e. selection of *m/z*-values which correspond to high and relevant peaks. The aim of the peak picking is to reduce the number of *m/z*-values by neglecting those values corresponding to noise signals or to non-specific baseline signals; for more on noise and baseline see [26], for more on the physical TOF model influencing the peak shape see [27], for more on statistical modelling of noise and baseline see [28]. Various peak picking methods for MALDI mass spectra are available and are implemented in mass spectrometry software packages. A recent comparison [29] shows that the methods which take into account the shape of a peak, and not just its intensity, perform the best. However, peak picking in MALDI-imaging poses new problems due to a large amount of spectra. Several approaches have been proposed. Firstly, the peak picking can be applied to the dataset mean spectrum. It is a very fast method and is implemented, e.g. in the ClinProTools software (Bruker Daltonik GmbH, Bremen, Germany). However, this method is not sensitive, since it does not favor high and relevant peaks presented only in a small part of a sample. For example, if a peak is present only in 1% of spectra (for an image of 100×100 pixels, this is an area of 10×10 pixels), then its contribution to the mean spectrum will be reduced by 100 times as compared to a low peak present in all spectra (e.g. a matrix peak). A consensus approach has been proposed [30], where among spectrum-wise picked peaks, those are selected, which are found in at least 1% of spectra. A similar approach, but requiring manual selection of regions of interest (ROIs) was proposed in [31]. In [30] and [32], for spectrum-wise peak picking, we applied the Orthogonal Matching Pursuit method which has complexity *O*(*n*^2^), where *n *is the length of a spectrum (usually 10,000-100,000). In general, one should consider efficient (at least *O*(*n*^2^)) peak picking methods when applied to MALDI-imaging data. Designing and performing a spectrum-wise peak picking, one should keep in mind an inherent balance between efficiency and sensitivity. Firstly, processing all spectra makes the method potentially more sensitive than processing just a part of the spectra. Secondly, the more peaks are selected per spectrum, the more sensitive the method can be. However, increasing sensitivity in both cases leads to longer processing times.

When constructing a list of dataset-relevant peaks out of the spectrum-wise peak lists, *m/z*-values selected in different spectra for the same peak can slightly differ. This effect cannot be completely compensated by the instrument calibration using reference markers (e.g. using a mixture of peptides with known molecular masses) and is caused by instrumental and experimental variation. In order to counterbalance this effect, a peak alignment procedure should be applied. Although the peak alignment is a well-known task in mass spectrometry, there are no dedicated studies of peak alignment in MALDI-imaging. Norris et al. briefly discuss peak alignment in the context of MALDI-imaging [24]. We have proposed an original but simple procedure for alignment of peaks with respect to the mean spectrum [32], another group reported the use of the Matlab (The Mathworks Inc., Natick, MA, USA) routine msalign [33].

### Unsupervised data mining

Most statistical learning methods can be divided into two groups, so-called unsupervised and supervised methods. Unsupervised methods are used for data mining, can be applied without any prior knowledge, and aim at revealing general data structure. Supervised methods (mainly classification) require specifying at least two groups of spectra which need to be differentiated, e.g. by finding *m/z*-values differentiating spectra of tumor regions from spectra of control regions. In the context of MALDI-imaging, two unsupervised approaches have obtained recognition: component analysis and spatial segmentation.

Component analysis represents a MALDI-imaging dataset with few score plots (or score images) and coefficients of contribution of each score image to each original *m/z*-image [34]. Mathematically speaking, a set of score images is a generating system of all *m/z*-images, that is, each *m/z*-image from the dataset can be represented as a sum of score images multiplied with respective coefficients. In the framework of MALDI-imaging, the most well-known component analysis method is the Principal Component Analysis (PCA) [34]. Other methods have been also studied: probabilistic latent semantic analysis [35], independent component analysis and non-negative matrix factorization [36]. For a recent comparison of component analysis methods, see [37].

#### Principal Component Analysis

**Figure 2:**
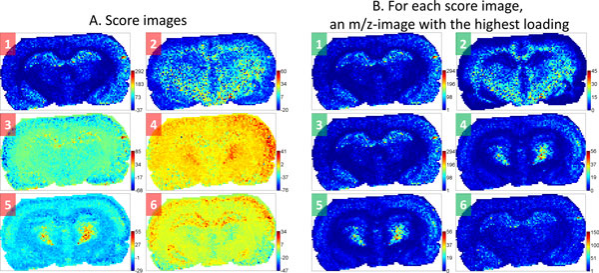
**PCA analysis of a MALDI-imaging dataset**. Illustration of shortcomings of PCA analysis of a MALDI-imaging dataset of a rat brain coronal section of 10 *μm *thickness (160 *μm *spatial resolution, 101×60 pixels, 5053 spectra). A. Score images of first six principal components (PC). B. For each score image, the *m/z*-image with the highest loading is plotted. One can see that the visual correlation between a score image (left) and its highest-loading *m/z*-image (right) is achieved only for PCs 1, 2, and 5, whereas not achieved for PCs 3, 4, and 6. Thus, the use of loadings for interpreting score images is not recommended.

In this section, we consider PCA which is the most well-known component analysis method used for MALDI-imaging data representation. PCA is a well-established statistical method and is often exploited for analysis, visualization, and compression of biological data. PCA and its variants [34] were early proposed for data mining in MALDI-imaging. For an illustrative tutorial on PCA for molecular biologists, see [38]. Using PCA, one can represent the full dataset with a few score images corresponding to first principal components. These score images reveal spatial structures hidden in the dataset by showing prominent spatial patterns (high intensity regions). However, except for showing the spatial patterns, the interpretation of score images provided by PCA is problematic. PCA score images can have negative values which are non-interpretable in terms of mass spectra intensities. Additionally, PCA score images do not define regions of interest and should be examined and interpreted visually. Finally, the way PCA is used currently (showing score images of first principal components and finding *m/z*-values of highest loadings) sometimes fails in selecting *m/z*-images co-localized with a score image. The *m/z*-images found using PCA sometimes look different from the corresponding score images; see Figure 2 for an illustration of this shortcoming. Some studies reported success in finding *m/z*-values using PCA [39], but they used PCA to discriminate two groups of *m/z*-values, each with unknown localization, rather than finding *m/z*-values for a specific spatial region. Deininger et al. [38] conclude that PCA is of use for data evaluation to decide "whether the experiment was successful or if preparation artifacts are present".

#### Spatial segmentation

**Figure 3:**
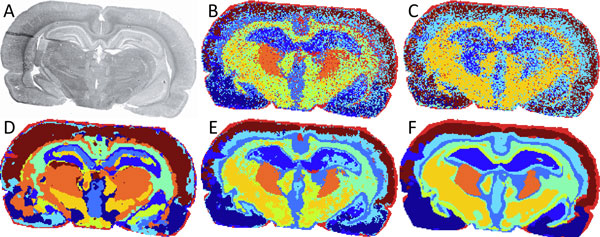
**Segmentation maps of the MALDI-imaging dataset**. Optical image (A) and segmentation maps (B-F) of the dataset from Figure 2 but with 80 *μm *spatial resolution. B. Straightforward k-means clustering of spectra. C. Hierarchical clustering (Euclidean distance, complete linkage) after PCA-reduction of spectra to 70% explained variance. D. Clustering after edge-preserving image denoising; moderate denoising, reprinted from [30] with permission from American Chemical Society. E-F. Efficient spatially-aware clustering, moderate size of data-adaptive neighborhood (E) and large size of non-adaptive neighborhood (F), reprinted from [52] with permission from Elsevier.

Spatial segmentation represents a MALDI-imaging dataset with one image, a segmentation map, where regions of distinct molecular composition are color coded, see examples in Figure 3. The spatial segmentation is performed by grouping all spectra by their similarity using a clustering algorithm. Then, all pixels are pseudo-color coded according to cluster assignment. Note that a color is assigned to a cluster, not to a distinct region; a segmentation map can have several spatially disconnected regions of the same color. Several advanced spatial segmentation methods have been proposed: hierarchical clustering with PCA used as preprocessing [38,40], and two methods suppressing the pixel-to-pixel variability which is inherent to MALDI-imaging: clustering with edge-preserving image denoising [30] and efficient spatially-aware clustering [32]. The last approach proposes a new spectral distance which accounts for spatial relations between spectra and presents an efficient distance-based method for finding segmentation where distances are computed on the fly.

**Hierarchical clustering **is advantageous providing clustering results in the form of a dendrogram which can be interactively analyzed. It is implemented in the flexImaging software (Bruker Daltonik) and was used in e.g. [39,40]; for a histopathological discussion see a recent review [20]. The main flaw of the hierarchical clustering is that it requires the distance matrix of size of *n×n *(*n *is the number of spectra) to be loaded into memory, that hinders processing of datasets with a large number of spectra. Moreover, it is subject to the pixel-to-pixel variability leading to noisy segmentation maps, see Figure 3. As for the parameters (distance, linkage) Deininger et al. [38,40] recommend choosing the Euclidean distance and the Ward linkage.

**Clustering suppressing pixel-to-pixel variability **has been recently proposed [30,32]. Both methods outperform hierarchical clustering by providing smooth, noiseless, and detailed segmentation maps. Although no publicly available implementations are provided yet, the second method [32] can be relatively easily implemented. For examples of segmentation maps produced with various methods, see Figure 3.

#### Interpretation of a segmentation map

**Figure 4:**
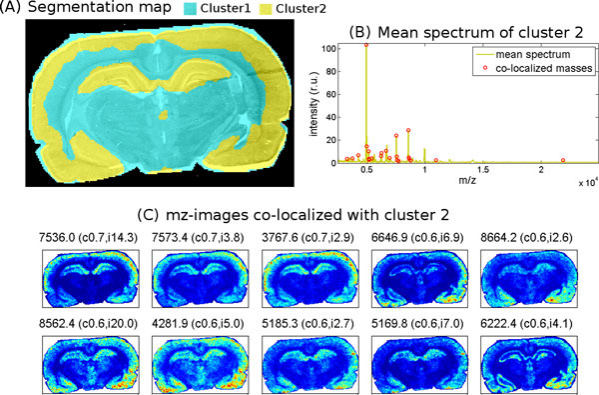
**Interpretation of a segmentation map of the MALDI-imaging dataset**. Spatial segmentation analysis of the MALDI-imaging dataset from Figure 2 with our algorithm from [30]. A. Segmentation map (two clusters) overlaid with the optical photo of the section. B. Mean spectrum of spectra from the second cluster. C. Pseudo-colored images of *m/z*-values spatially co-localized with the second cluster; each title shows the *m/z*-value, Pearson correlation between the image and the cluster spatial map, and 90%-quantile image intensity.

In contrast to PCA, spatial segmentation maps not only elucidate the spatial structure of the dataset, but can be easily interpreted in terms of *m/z*-values associated with a specific part of revealed spatial structure. Each segmentation map consists of a given number of clusters, each represented with its pseudo-color. After a visual examination, if a cluster represents a region of interest, then the associated *m/z*-values can be found as proposed in [30]. A spatial mask corresponding to the selected cluster is considered and for each *m/z*-image its correlation with this mask is calculated. Finally, co-localized *m/z*-values with highest and significant (*p*-value smaller than 0.05) correlation should be considered. An illustration is given in Figure 4. So far, this simple but powerful method is not implemented in major MALDI-imaging software packages and requires an in-house implementation. Other more complicated methods using spatial querying [41,42] have been proposed; the software package presented in [41] is publicly available.

#### High-throughput pipeline for interpretation of MALDI-imaging data using spatial segmentation

**Figure 5:**
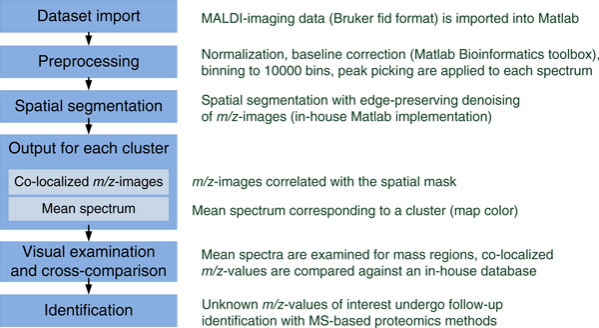
**Pipeline for interpretation of a MALDI-imaging dataset**. Our pipeline for interpretation of a MALDI-imaging dataset using spatial segmentation.

Here, we present our pipeline for interpretation of a MALDI-imaging dataset using spatial segmentation which was successfully applied to hundreds of MALDI-imaging datasets at the Dorrestein Lab, University of California San Diego; see Figure 5. The characterization of natural products of bacteria was the main subject of these studies, see e.g. [43], which involved analysis of pairwise interactions of many bacterial species under different conditions. Our pipeline was able to process up to a few hundreds of MALDI-imaging datasets per week, representing the results in a concise way so that a few tens of datasets a week could be easily interpreted by one scientist. Our results were computed and, more importantly, interpreted in a time comparable with the dataset acquisition time. In contrast, a manual analysis of a single MALDI-imaging dataset takes days and, as we found, is still not as exhaustive and sensitive as the automatic analysis.

Based on our experience in developing and applying the MALDI-imaging data analysis pipeline, the following recommendations can be made. It is of crucial importance to represent the data in the most understandable and compact way for a biologist or practitioner, otherwise large amount of information extracted out of a MALDI-imaging dataset will not be appreciated. Providing a segmentation map is only a part of data analysis process. Interpretation of the segmentation map is as (or even more) important as the segmentation itself. When finding co-localized *m/z*-values based on a segmentation map, one should consider all *m/z*-values but not only those selected by a peak picking. Selecting too many peaks during the peak picking prior to segmentation is not always needed, often detailed segmentation does not need many peaks. Selecting many peaks slows down the segmentation and can introduce additional variation; usually 50-200 peaks is a good choice, although it depends on the analyzed mass range and samples. Memory requirements of a processing algorithm can be more important than the computational efficiency because the available memory is limited whereas the number of spectra increases quadratically with increasing the spatial resolution. One should consider memory-efficient methods which have *O*(*n*) memory requirements (*n *is the number of spectra) and ideally do not require storing the full dataset in the memory. Once a MALDI-imaging pipeline is developed and tested, it should be integrated with other computational tools for mass spectrometry analysis, that requires at least providing export of all valuable information into common format.

### Supervised classification

In this section we consider how supervised classification can be used for biomarker discovery. Classification requires specifying at least two groups of spectra and aims at differentiating these groups. Let us consider the task of cancer biomarker discovery which involves comparison of tumor and control regions of a biopsy tissue. One can also compare several tumor sections versus several control sections, collected from one or several patients. A classification algorithm, the so-called classifier, considers two groups of spectra and undergoes training to be able to discriminate the groups of spectra. If the training was successful that can be confirmed by a high classification accuracy (also called as the correct rate or the recognition rate) close to 100%, then one could apply the classifier to new spectra to determine their class (tumor or control), like in [44,45]. However, in biomarker discovery studies one is interested not only in application of the classifier to new spectra, but in interpreting the differences between the tumor and control groups of spectra which were found by the classifier, namely, in the tumor-discriminative *m/z*-values. Later on, molecular identities of these tumor-discriminative *m/z*-values can be established using MS-based proteomics methods.

Currently, classification of MALDI-imaging spectra for the search of biomarkers is an active area of research. Lemaire et al. [46] used the StatView 5.0 software (SAS Institute, Cary, NC) with symbolic discriminant analysis and statistical tests for the search for a new ovary cancer biomarker. Groseclose et al. [47] used the ClinProTools software (Bruker Daltonik) with the support vector machine algorithm to differentiate adenocarcinoma from squamous cell carcinoma. Cazares et al. [48] used ClinProTools with the genetic algorithm and the SAS 9.1 statistical software (SAS Institute) to discriminate prostate cancer. Rauser et al. [20] used the R statistical package (http://www.r-project.org) with the support vector machine and artificial neural network algorithms for classification of HER2 receptor status in breast cancer tissues.

However, in all above cited studies, the classification methods developed for conventional MALDI mass spectrometry were used, which do not take into account specifics of MALDI-imaging data. Classification methods for MALDI-imaging data are still to be developed. Here, we give several recommendations on the most important points to consider when applying classification to MALDI-imaging data.

Firstly, the compared groups are often imbalanced, that is, they have significantly different sizes. Classification of imbalanced data requires special classification and evaluation methods, otherwise the classification can be biased towards a larger group. This issue is well-studied, and advanced methods for its solution were proposed [49-51]. In our experience, large number of spectra in MALDI-imaging normally allows one to compensate moderate imbalance (up to ten-fold) by simple decimation of the larger group. Namely, we consider only each *k*-th spectrum of the larger group, where *k *should be adjusted to achieve the balance between groups sizes. However, for compensating a strong imbalance, advanced methods (e.g. sampling and cost-sensitive learning) are recommended, see [49-51].

Secondly, although classification of conventional dried droplets MS data is evaluated by how close the classification accuracy is to 100%, one should not aim at achiving this theoretically highest possible accuracy in classification of MALDI-imaging spectra for the following reasons. MALDI-imaging spectra show significant heterogeneity because of technical reasons (noise, tissue mixture at the available spatial resolution, ions diffusion). Moreover, one cannot expect the annotation of a tumor region to be of perfect quality because of manual mistakes and a lack of the expert time. Additionally, the annotation does not go down to the cellular or subcellular level, where real differentiation between cells takes place. All this leads to classification accuracies lower than 100%. However, if a classifier produces a low accuracy (close to 50% for balanced groups), this indicates some problems and the provided discriminative *m/z*-values should be considered with caution. In our experience, the good accuracy values above 80%.

Thirdly, the discriminative *m/z*-values provided by the classification should always be visualized as *m/z*-images and manually examined whether their spatial patterns are relevant (e.g. co-localized with the tumor area). MALDI-imaging provides a unique way of evaluating the relevance of *m/z*-values by their spatial pattern, that should be done before starting tedious identification of molecular identities of putative biomarkers.

### Visualization of *m/z*-images

A computational analysis of a MALDI-imaging dataset, either using unsupervised methods or using supervised classification, delivers a list of *m/z*-values of interest. In order to associate these *m/z*-values with their molecular identities, one needs to perform their identification, usually with MS-based proteomics methods. Before starting identification, one usually examines provided *m/z*-values comparing them with the *m/z*-values known in the field. If the list contains *m/z*-values related to each other in a known manner, this increases the confidence in that they express biologically relevant information. For example, a few *m/z*-values separated by one unit can correspond to isotopes (in MALDI, ions usually have a charge of +1). Two *m/z*-values separated by 17 units can correspond to the same compound before and after the loss of ammonia. The difference of 18 units corresponds to the loss of water. The difference of 16 units corresponds to oxidation of methionine (or another amino acid side chain). Finally, *m/z*-values of interest undergo identification.

**Figure 6:**
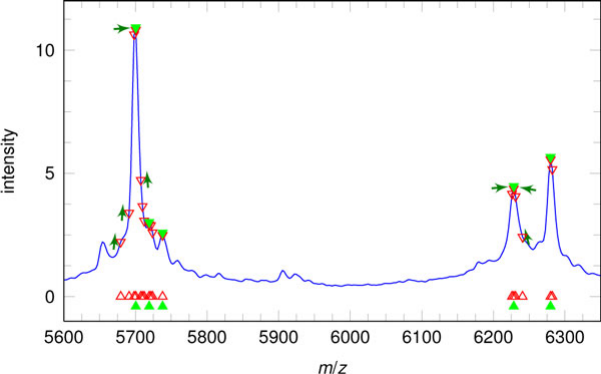
**A method of alignment of selected *m/z*-values**. A method of alignment of selected *m/z*-values using the dataset mean spectrum. The mean spectrum is shown in blue. Red triangles indicate *m/z*-values of interest before the alignment. Green arrows illustrate the process of alignment. Green triangles show aligned peaks and their *m/z*-values. Reprinted from [52], Copyright 2011, with permission from Elsevier.

Usually, a computational analysis can deliver a long list of masses, and a simplification and shortening of this list by not loosing the sensitivity of the automatic processing is an important task. In the context of MALDI-imaging, one method, called masses alignment, was proposed by us [32] and successfully tested in another study [52]. The main idea of this method is to group masses corresponding to one peak and then represent them with one *m/z*-value. For this purpose, we use the dataset mean spectrum and align the selected *m/z*-values, "moving them" uphill the dataset mean spectrum so that they merge into the local maxima of the mean spectrum; see Figure 6 for an illustration. This method allowed us to reduce the number of *m/z*-values without loss of information.

**Figure 7:**
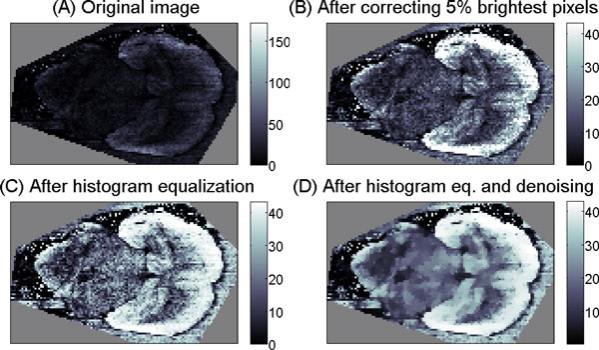
**Improving visualization of an *m/z*-image**. Improving visualization of an *m/z*-image by contrast-enhancement and image denoising. A. An *m/z*-image from a MALDI-imaging dataset for a transverse section of mouse brain. B. After contrast-enhancement by correcting 5% of the brightest pixels. C. After contrast-enhancement by histogram equalization. D. After histogram equalization and edge-preserving denoising. Reprinted from [2] with permission from John Wiley and Sons.

Once *m/z*-values are provided by a computational analysis, their *m/z*-images should be examined in order to visually correlate their spatial patterns with known spatial features of the sample. A usual MALDI-imaging study results in many *m/z*-images and, as we demonstrated in [2], the problem of their visualization remains important. Recall that an *m/z*-image is a real-valued image showing mass spectra intensities at the given *m/z*-value. Usually, one visualizes an *m/z*-image using a pseudo-color scale, assigning gradually changing colors to the intensities. The first problem faced when using this visualization is the so-called hot spots, that is separate pixels or small groups of pixels with artificially high intensities. Such pixels distort the pseudo-color scale so that other pixels are shown with insufficient contrast. In order to automatically correct the hot spots, we proposed [2] to suppress 5% of brightest pixels or to use an advanced contrast-enhancing procedure like histogram equalization, see Figure 7B-C for an illustration.

The second problem of visualization of *m/z*-images is the strong pixel-to-pixel variation which is inherent to MALDI-imaging technique. In [30], we analyzed this variation and showed that it has multiplicative nature with respect to the pixels intensity. That is, the higher the intensity in some spatial region, the stronger the noise in this region, which distorts the *m/z*-image and hampers visual evaluation of prominent features. In order to reduce this variability and suppress the noise, we proposed to apply image denoising to an *m/z*-image prior to visualization. Figure 7D illustrates application of advanced edge-preserving image denoising from [30].

## Current computational challenges

In this section, we consider current challenges associated with the statistical analysis of MALDI-imaging data. We hope that this discussion will be of interest to bioinformaticians and statisticians fostering computational research in this area.

### Available software

The commercially available software for MALDI-imaging delivered by mass spectrometry vendors is aimed at data acquisition and does not provide capabilities for statistical analysis yet. Bruker Daltonik (Bremen, Germany) delivers flexImaging (visualization) and, optionally, ClinProTools (multivariate analysis, PCA, classification) which however can be used for small datasets only. Thermo Scientific (Waltham, MA, USA) provides ImageQuest (visualization). Waters (Manchester, UK) provides HDI Software (visualization) which can be coupled with MassLynx (peak picking) and MarkerLynx (PCA, orthogonal projection least squares), although no publications involving MarkerLynx are known yet. Shimadzu (Nakagyo-ku, Kyoto, Japan) provides Intensity Mapping (visualization, export). In addition to vendor-provided software, Novartis (Basel, Switzerland) provides the BioMap software which can be used for visualization and calculating basic statistics of the full dataset or of regions of interest. AB Sciex (Foster City, CA, USA) provides TissueView which is based on the BioMap software. Currently, in-house developments are necessary and Matlab is probably the most popular development and computing environment in the MALDI-imaging field.

### Specific challenges of analyzing MALDI-imaging data

Two general considerations proved to be important in our practice when developing methods for processing MALDI-imaging data. Firstly, a MALDI-imaging dataset is large, that requires computational methods to be runtime and memory efficient. A typical dataset is comprised of 5,000-50,000 spectra, each having 10,000-100,000 intensity values. Datasets generated using upcoming high spatial resolution and high mass resolution MALDI-imaging techniques (e.g. MALDI-FT-ICR-imaging) or using 3D MALDI-imaging are several fold larger. At the same time, the first examination of acquired data is usually done on a workstation attached to the mass spectrometer. Processing single datasets on the same workstation is desirable, that imposes additional constraints regarding memory demands and computational costs. Ideally, the processing time should not exceed the acquisition time which is a few hours for a typical MALDI-imaging dataset. Secondly, MALDI-imaging data suffers from the strong pixel-to-pixel variation which can be significantly suppressed by using methods respecting spatial relations between pixels. As demonstrated by us, performing image denoising prior to clustering [30,41] or considering each spectrum together with its spatial neighbors [32] leads to smoother and more detailed results. The advantage of respecting spatial relations between spectra was demonstrated for other problems as well [53].

Statistical modelling of pixel-to-pixel variability could help developing processing methods. However, this, as well as modelling of other statistical effects in MALDI-imaging data (noise, baseline generation, variability in the shape of a peak), is a scarcely studied field. Although a physical model of the time of flights distribution for MALDI-TOF mass spectrometry was proposed already in 2005 [27], a little progress is seen since then. The problem of statistical modelling for MALDI-imaging data is addressed only marginally [30]. Successful modelling of this data would provide a way of evaluation of computational methods by using simulated data. Additionally, the statistical modelling can be used for development of computational methods taking into account the statistical models, e.g. model-based classification methods or statistical image processing, as it was illustrated for SIMS data processing [54].

### Quality assurance

Quality assurance for MALDI-imaging data is not developed yet. There exist no standard operation procedures for estimating the quality of a full dataset or single spectra. We have recently proposed a visualization method for a quick quality check [2], but there is a lot to be done in this area. Automatic quality evaluation of single spectra of a MALDI-imaging dataset is of special importance, since, due to biochemical complexity of a sample, and various weakly studied effects of matrix allocation and MALDI ionization, some spectra show artificial patterns leading to hotspots and distorting computational analysis. Such artificial spectra could be detected and removed by methods of outliers detection developed specifically for MALDI-imaging.

### Noise-tolerant statistical learning

When preparing a training set of spectra in a MALDI-imaging biomarker discovery study, the annotation is normally done by a visual examination of a sample and by a manual annotation of regions representing different classes (e.g. tumor and control). However, due to the rough character of this annotation, and due to inherent chemical complexity on the scale resolved by MALDI-imaging, the annotation can be incorrect for a significant portion of spectra. For instance, some pixels in the region annotated as a control one, can contain tumor cells. In statistical learning, this effect is referred to as classification noise or noise in labels [55]. When classifying spectra of a MALDI-imaging dataset, classification methods tolerating classification noise or, in general, methods with high generalizability should be considered.

### Combination with other 2D imaging modalities

Combination of MALDI-imaging and microscopy images of stained tissue used in immunohistochemistry can be used for improvement of MALDI-imaging data analysis. This approach is of special importance because the spatial resolution of MALDI-imaging is lower than of microscopy and the pixel-to-pixel variability is significantly stronger. Implementation of this approach requires special co-registration methods.

## Competing interests

The author declares that he has no competing interests.

## Authors' contributions

TA wrote the manuscript and performed data analysis.
